# Hazardous alcohol consumption across different industries in Sweden: a pooled cross-sectional study

**DOI:** 10.1093/alcalc/agae077

**Published:** 2024-11-11

**Authors:** Emelie Thern, Katrina J Blindow, Erica Jonsson, Emma Brulin, Jonas Landberg, Theo Bodin, Devy L Elling

**Affiliations:** Institute of Environmental Medicine, Karolinska Institute, Solnavägen 4, 113 65 Stockholm, Sweden; Department of Public Health Sciences, Stockholm University, 106 91 Stockholm, Sweden; Institute of Environmental Medicine, Karolinska Institute, Solnavägen 4, 113 65 Stockholm, Sweden; Institute of Environmental Medicine, Karolinska Institute, Solnavägen 4, 113 65 Stockholm, Sweden; Institute of Environmental Medicine, Karolinska Institute, Solnavägen 4, 113 65 Stockholm, Sweden; Department of Public Health Sciences, Stockholm University, 106 91 Stockholm, Sweden; Department of Clinical Neuroscience, Karolinska Institute, 171 65 Stockholm, Sweden; Institute of Environmental Medicine, Karolinska Institute, Solnavägen 4, 113 65 Stockholm, Sweden; Centre for Occupational and Environmental Medicine, Region Stockholm, 113 65 Stockholm, Sweden; Institute of Environmental Medicine, Karolinska Institute, Solnavägen 4, 113 65 Stockholm, Sweden

**Keywords:** alcohol, industry, health, work environment, nicotine use

## Abstract

**Aim:**

The current study aims to (i) examine differences in hazardous alcohol consumption across different industries in Sweden and (ii) assess to what degree any such difference can be attributed to a differential distribution of nicotine use, health, and work environments among individuals working in these industries.

**Methods:**

A pooled cross-sectional study was conducted including all participants of the survey of Health, Work Environment, and Lifestyle Habits between 2012 and 2023 (*n* = 54 378), collected by an occupational health service company (Feelgood). The survey contained self-reported information on alcohol use, industry, nicotine use, health, and work environment. Crude and adjusted odds ratios with 95% confidence intervals were obtained by pooled logistic regression analyses.

**Results:**

Hazardous alcohol use was highly prevalent in the current study population (37%), especially among individuals in the accommodation/food service, arts/entertainment/recreation, and the construction industry. Compared to individuals working in education, individuals in these industries had >1.6-fold increased odds of reporting hazardous alcohol consumption. Differences in nicotine use and physical work environment between the industries explained some of the differences in hazardous alcohol consumption between industries, while differences in health and psychosocial work environment had limited effects on the estimates.

**Conclusion:**

We identified several industries in the Swedish workforce where hazardous alcohol use is highly prevalent. While differences in nicotine use, health, and work environment explained a part of these risk differences, most of the risk differences remained.

## Introduction

In Sweden, ~80% of the adult population work, and a significant share (almost 20%) of these workers report hazardous use of alcohol (Myndigheten för arbetsmiljökunskap 2023). Hazardous drinking refers to a quantity or pattern of alcohol consumption that puts individuals at risk for adverse health events (Reid et al. 1999). In contrast, harmful drinking specifically results in adverse outcomes, such as physical or psychological harm (Reid et al. 1999). The consequences related to alcohol consumption entail significant costs for societies. In Sweden, these costs were estimated to be just over 100 billion SEK, and sickness absence was one of the main costs (Ramboll 2019). The relationship between alcohol consumption and work is complex due to its bidirectionality. On the one hand, work-related factors can influence alcohol consumption, for example, when alcohol is used to cope with work stress (Henkel 2011). On the other hand, alcohol consumption can contribute to work-related problems, such as workplace accidents leading to injuries, short- and long-term sickness absence, absenteeism, and decreased work performance (Thørrisen et al. 2019; Hashemi et al. 2022). Gainfully employed individuals spend a significant amount of their time at work, which makes the workplace a pertinent arena for identifying prevalence and predictors of hazardous alcohol consumption to inform effective prevention.

Most research looking into the role of work on alcohol consumption has mainly focused on looking into occupational differences, where less is known about the potential differences across industries (Roche et al. 2015). Occupational classifications organize individuals according to the specific jobs and tasks they perform, while industry classifications categorize individuals based on the economic sector in which they are employed (Mannetje and Kromhout 2003). The same occupation can, therefore, be found in several different industries such as project managers, accountants, human resource specialists, and administrative analysts. Subsequently, looking across industries could better reflect the general work context and alcohol norms in the workplace. Previous research suggests that certain industries, especially male-dominated and manual work, report a higher prevalence of hazardous and harmful alcohol consumption and related harm (Probst et al. 2014; Jones et al. 2015; Roche et al. 2015; Pulido et al. 2017; Thern and Landberg 2021; Thompson and Pirmohamed 2021). Most of the previous research focusing on occupational or industry differences in hazardous alcohol use has used broad occupational categories as indications of socioeconomic position or focused on certain industries known to be at high risk as opposed to examining the differences across a wide range of industries (Zhu et al. 2011; Probst et al. 2014; Roche et al. 2015; Tynan et al. 2017). This could be a limitation as it does not provide a nuanced picture of potential differences in alcohol consumption and related harm across different occupations or industries. To date, only a few studies have investigated the relationship between work and alcohol consumption by looking more specifically across industries (Zhu et al. 2011; Roche et al. 2015; Pulido et al. 2017; Tynan et al. 2017).

Hazardous alcohol consumption is often a result of a complex interplay between individual, social, and work-related factors. Thus, to enhance our understanding of the potential industry differences in hazardous alcohol use, it is important to explore how differences in individual and work-related factors contribute to this association. Given that age, sex, and nicotine use, as well as poor mental and general health, are all associated with hazardous alcohol consumption and related harm (Jane-Llopis and Matytsina 2006; WHO 2018; Van Nhat et al. 2024), a plausible assumption could be that the increased risk for hazardous alcohol use found in certain industries may be due to differences in individual-level risk factors of hazardous alcohol use. Still, only a few studies have explored whether differences in various risk factors can account for the industry disparities observed in hazardous alcohol use (Pulido et al. 2017; James et al. 2021). A recent Spanish study that investigated the contribution of individual-level factors on the association between industry and alcohol-related mortality found that age–sex adjustments explained a large proportion of the increased risk in industries dominated by men and older employees (Pulido et al. 2017). Furthermore, a large proportion of the well-known gradient in health by occupation has previously been explained by self-selection into occupations (Volkers et al. 2007; Ravesteijn et al. 2018).

Another potential mechanism is that certain work-related factors that are inherently more common in certain industries may themselves be associated with hazardous alcohol use. However, research looking at differences in work-related factors to explain industry differences in hazardous alcohol use is scarce. Previous research suggests that social support, job satisfaction, and decision latitude promote lower levels of alcohol intake (Roche et al. 2015). On the contrary, heavy workload, job insecurity, poor psychosocial work environment, and hostile work environment have been associated with an increased risk of hazardous alcohol consumption (Zhu et al. 2011; Heikkilä et al. 2012; Roche et al. 2015; Tynan et al. 2017; Almroth et al. 2022; Blindow et al. 2023). A study looking specifically at the mining industry in Australia found that differences in workplace factors and attitudes only explained a small proportion of the differences in hazardous alcohol consumption (Tynan et al. 2017). This is an important finding that needs to be further investigated in other industries and a different context with different alcohol norms and alcohol policies compared to Australia.

Thus, the current study aims to (i) examine differences in hazardous alcohol consumption across different industries in Sweden and (ii) assess to what degree any such difference can be attributed to a differential distribution of nicotine uses, health, and work environments among individuals working in these industries.

## Materials and methods

### Data source and study population

This pooled cross-sectional study was based on all individuals who have participated in Feelgood’s (an occupational health service company in Sweden) annual survey of Health, Work Environment, and Lifestyle Habits (HALU) between 2012 and 2023 (*n* = 93 854). If an individual participated multiple times in HALU, we included them only the first time they participated.

In Sweden, ~60% of all employees have access to an occupational health service (Arbetsmiljöverket 2022). Individuals who are self-employed, employed in small enterprises, temporarily employed, or working in certain industries, such as accommodation and food service activities, are less likely to have access to occupational health services (Arbetsmiljöverket 2022). Access is provided through a reimbursement agreement between the organization/employer and the occupational health service. Feelgood has customers from several organizations across various industries, both in the private and public sectors, which collectively entail ~1.2 million employees (Feelgood 2023). According to Feelgood, the response rate for the HALU survey has been ~70% between 2012 and 2017. From 2017, it has gradually decreased to ~50% in 2023.

In the current study, individuals were excluded if (i) they had missing information on the questions related to alcohol (*n* = 12 495), (ii) they had missing information on industry (*n* = 26 972), or (iii) they had been working in the activities of extraterritorial organizations industry (*n* = 9), as shown in Supplementary Fig. 1. In general, the excluded individuals had slightly worse health and a slightly better work environment compared to the included individuals (Supplementary Table 1). The final analytical sample consisted of 54 378 respondents.

### Variables

#### Exposure: industry classification

Self-reported information on the exposure variable was obtained from the HALU survey. Respondents were asked to specify the name of the company they were currently employed in. This information was used by Feelgood to define their industry according to the Swedish Standard Industrial Classification (SNI) (SCB 2023), which is equivalent to the European NACE2 (European Communities 2008). The SNI classifies businesses and workplaces according to the activity carried out. In the current study, the industry classification was categorized based on the first level of the SNI 2007, as shown in Table 1.

**Table 1:** Characteristics of the study population

Industries	Total	Female (%)	Age (mean ± sd)	Nicotine use (%)	Poor general health (%)	Feeling depressed (%)	Feeling anxious (%)	Low work satisfaction and engagement (%)	Low social support (%)	High workload (%)	Workplace violence (%)	Heavy lifting (%)
Accommodation and food service activities	126 (0.2)	72 (57.1)	39.3 ± 11.5	50 (39.7)	5 (4.0)	5 (4.0)	4 (3.2)	42 (33.3)	39 (31.0)	21 (16.7)	6 (4.8)	74 (58.7)
Arts, entertainment, and recreation	393 (0.7)	224 (57.0)	43.7 ± 11.1	90 (23.1)	18 (4.6)	19 (4.8)	30 (7.6)	134 (34.1)	113 (28.8)	96 (24.4)	4 (1.0)	96 (24.4)
Construction	13,880 (25.7)	2083 (15.0)	38.3 ± 12.7	5736 (41.8)	542 (3.9)	304 (2.2)	410 (3.0)	3645 (26.3)	3296 (23.8)	1822 (20.5)	140 (1.0)	7146 (51.5)
Financial and insurance activities	2645 4(4.9)	1336 (50.5)	40.5 ± 11.2	531 (20.2)	91 (3.4)	80 (3.0)	104 (3.9)	705 (26.7)	519 (16.6)	455 (17.2)	16 (0.6)	20 (0.8)
Administrative and support service activities	1215 (2.3)	484 (39.8)	41.7 ± 10.5	381 (31.7)	48 (4.0)	44 (3.6)	56 (4.6)	401 (33.0)	299 (24.6)	264 (21.7)	6 (0.5)	338 (27.8)
Wholesale and retail trade	5368 (9.9)	1552 (28.9)	41.9 ± 1.4	1746 (32.8)	226 (4.2)	153 (2.9)	204 (3.8)	1624 (30.3)	1432 (26.7)	1101 (20.5)	19 (0.4)	1645 (30.6)
Water supply; sewerage, waste management, and remediation activities	523 (1.0)	140 (26.8)	42.7 ± 11.8	198 (38.2)	15 (2.9)	13 (2.5)	17 (3.3)	149 (28.5)	133 (25.4)	65 (12.4)	25 (4.8)	213 (40.7)
Professional, scientific, and technical abilities	6505 (12.0)	2841 (43.7)	39.6 ± 11.1	1285 (19.9)	235 (3.6)	165 (2.5)	252 (3.9)	1707 (26.2)	1542 (23.7)	1191 (18.3)	20 (0.3)	554 (8.5)
Transportation and storage	2086 (3.9)	870 (41.7)	43.2 ± 11.1	604 (29.2)	91 (4.4)	59 (2.8)	99 (4.8)	718 (34.4)	636 (30.5)	432 (20.7)	20 (1.0)	404 (19.4)
Information and communication	2893 (5.4)	995 (34.4)	43.4 ± 10.2	596 (20.8)	114 (3.9)	83 (2.9)	114 (3.9)	853 (29.5)	716 (24.8)	568 (19.6)	22 (0.8)	156 (5.4)
Manufacturing	5972 (11.0)	1294 (21.7)	44.1 ± 11.9	1947 (33.1)	250 (4.2)	151 (2.5)	167 (2.8)	2127 (35.6)	1627 (27.2)	1002 (16.8)	24 (0.4)	2274 (38.1)
Real estate activities	2470 (4.6)	1079 (43.7)	43.9 ± 11.1	640 (26.0)	110 (4.5)	70 (2.8)	98 (4.0)	553 (22.4)	553 (22.4)	450 (18.2)	77 (3.1)	592 (24.0)
Electricity, gas, steam, and air conditioning supply	558 (1.0)	104 (18.6)	44.4 ± 11.1	186 (33.4)	22 (3.9)	14 (2.5)	14 (2.5)	139 (24.9)	130 (23.3)	95 (17.0)	4 (0.7)	193 (35.6)
Public administration and defence, compulsory social security	3048 (5.6)	1991 (65.3)	45.4 ± 11.0	555 (18.4)	122 (4.0)	109 (3.6)	136 (4.5)	703 (23.1)	687 (22.5)	569 (18.7)	154 (5.1)	510 (16.7)
Mining and quarrying	161 (0.3)	38 (23.6)	44.8 ± 13.9	73 (45.3)	3 (1.9)	2 (2.5)	4 (2.5)	64 (40.0)	45 (28.0)	11 (6.8)	1 (0.6)	10 (6.2)
Other service activities	2095 (3.9)	1270 (60.6)	46.0 ± 11.6	378 (19.2)	94 (4.5)	89 (4.3)	106 (5.1)	414 (19.8)	472 (22.5)	308 (14.7)	60 (2.9)	366 (17.5)
Education	2154 (4.0)	1103 (51.2)	43.5 ± 12.2	266 (12.4)	93 (4.3)	109 (5.1)	160 (7.4)	498 (23.1)	635 (29.5)	492 (22.8)	15 (0.7)	153 (7.1)
Human health and social work activities	2106 (3.9)	1594 (75.7)	46.3 ± 11.3	380 (18.1)	155 (7.4)	123 (5.8)	143 (6.8)	477 (22.7)	490 (23.3)	508 (24.1)	182 (8.6)	615 (29.2)
Agriculture, forestry, and fishing	180 (0.3)	37 (20.1)	41.1 ± 13.5	58 (32.2)	8 (4.4)	4 (2.2)	5 (2.8)	35 (19.4)	26 (14.4)	18 (10.0)	2 (1.1)	72 (40.0)

#### Outcome: hazardous alcohol use

The outcome of hazardous alcohol use was measured using the first three questions of the Alcohol Use Disorders Identification Test (AUDIT-C). The AUDIT-C includes questions related to an individual’s alcohol consumption, including the following questions ‘How often did you have a drink containing alcohol in the past year?’, ‘How many drinks did you have on a typical day when you were drinking in the past year?’ and ‘How often did you have six or more drinks on one occasion in the past year?’. Respondents were asked to respond to each question on a 5-point Likert scale, which was then summarized to a scale of 0–12 (0 indicating no alcohol use). In line with general recommendations in Sweden for AUDIT-C, men who scored 5 points or higher and women who scored 4 points or higher on the summarized scale were considered to have the outcome of hazardous alcohol consumption (Berman et al. 2012).

#### Potential explanatory factors

##### Sociodemographic

Self-reported sociodemographic information on sex (male or female) and age was obtained.

##### Nicotine use

Self-reported information on smoking and snuff use was obtained. Individuals reporting either currently smoking or currently taking snuff were considered as using nicotine.

##### Health

Several self-reported measures of health were derived from the survey to cover mental health and general health. Respondents were asked to respond on a 4-point Likert scale ranging from 0 (never) to 3 (often) on questions related to feeling anxious (‘I feel strong anxiety and/or worry’,) and feeling depressed (‘I feel depressed’.). Individuals reporting ‘often’ having anxiety or ‘often’ feeling depressed were considered as feeling depressed or anxious. On a 5-point Likert scale (ranging from very poor to very good), respondents were asked to evaluate their perceived health (‘How do you perceive your health?’). Individuals reporting ‘very poor’ or ‘poor’ perceived health were considered as having poor general health.

##### Psychosocial work environment

The HALU survey included 21 questions about the psychosocial work environment. Each question included a 4-point Likert scale, with responses ranging from 0 (never) to 3 (often). We conducted a principal component analysis including all items resulting in a three-factor solution (data showing evidence for the three-factor solution, see Supplementary Table 2 for more details on the items included in each factor). The first factor was ‘work satisfaction and engagement’, which included nine different items covering dimensions such as meaningfulness, engagement, stimulation, control, role clarity, and skill development. After summing up the nine items, quartiles were calculated, and the individuals in the lowest quartile were considered as having low work satisfaction and engagement. The second factor was ‘social support’, which included six items covering dimensions of collaboration, support, appreciation, and respect. The six items for ‘social support’ were summed up, and quartiles were calculated. Individuals in the lowest quartile were considered to have low social support. The last factor was ‘workload’, which encompassed six items covering dimensions such as stress, demands, urgency, and time pressure. The items were summed together, and then, quartiles were calculated. Individuals in the highest quartile were defined as having a high workload. All factors demonstrated high internal consistency (Cronbach’s alpha of 0.83–0.86) (Cortina 1993). Furthermore, respondents who answered yes to ‘I am exposed to violence or threats of violence at work’ were considered exposed to workplace violence.

##### Physical work environment

Self-reported information about the physical work environment was collected. Respondents who answered yes to ‘I have heavy lifting in my job’ were considered to have a physically demanding job.

### Statistical analysis

Pooled logistic regression models were used to investigate the association between industry and hazardous alcohol consumption. The estimates were expressed as crude and adjusted odds ratios (ORs) with 95% confidence intervals (CI). In line with previous research, the education industry was set as the reference group (Pulido et al. 2017).

First, a crude model estimating the association between industry and hazardous alcohol consumption was carried out. Next, adjustments for the year of participating in HALU and sociodemographic covariates were performed (Model 1). Then, a series of models with additional separate adjustments for nicotine use (Model 2), health (Model 3), psychosocial work environment (Model 4), and physical work environment (Model 5) was conducted. All potential explanatory factors were entered simultaneously in the final model (Model 6).

To check the robustness of our results, several sensitivity analyses were performed. First, individuals who participated during the pandemic (between 2020 and 2021) were excluded (*n* = 5515, 10.1%) as the response rate was much lower at this time compared to the other years (Feelgood 2023). Second, respondents who had indicated they were on sick leave for more than four consecutive weeks in the past year were excluded (*n* = 2445, 4.5%) since sickness absence is a major risk factor for hazardous alcohol use (Amiri and Behnezhad 2020). Lastly, a higher cut-off for the AUDIT-C, 5 points for women and 6 points for men, was implemented in an additional analysis to investigate the association between industry and alcohol use disorder (AUD) (Lundin et al. 2015).

Sensitivity analyses using imputed data to handle the large number of missing values on key variables such as industry and hazardous alcohol use were performed, demonstrating similar results as the one presented below (Supplementary Table 3). As the same conclusions were reached when excluding observations with missing data, missing values for the potential explanatory variables were coded as separate categories (Supplementary Table 4). All analyses were performed using Stata Statistical Software, release 17.

## Results

Table 1 shows the characteristics of the study population. Many individuals in the current study came from the construction (25.7%) and professional/scientific/technical abilities (12.0%) industries. Less than 1% of the study population came from either the accommodation/food industry, arts/entertainment/recreation industry, the mining/ quarrying industry, or the agriculture/forestry/fishing industry. There was a greater proportion of males (64.9%) compared to females (35.1%) in the current study. In general, there was a higher prevalence of nicotine use in male-dominated industries (Table 1). Less than 5% experienced poor general health, feelings of depression, or anxiety. A poor psychosocial work environment was more often reported by individuals working in industries such as arts/entertainment/recreation, administrative/support service activities, manufacturing, mining/quarrying, and transportation/storage. Workplace violence was, to a greater degree, reported by individuals in the public administration/defence/compulsory social security industry, as well as the human health/social work industry. Physically demanding work was more common in the construction and agriculture/forestry/fishing industry.

**Figure 1:**
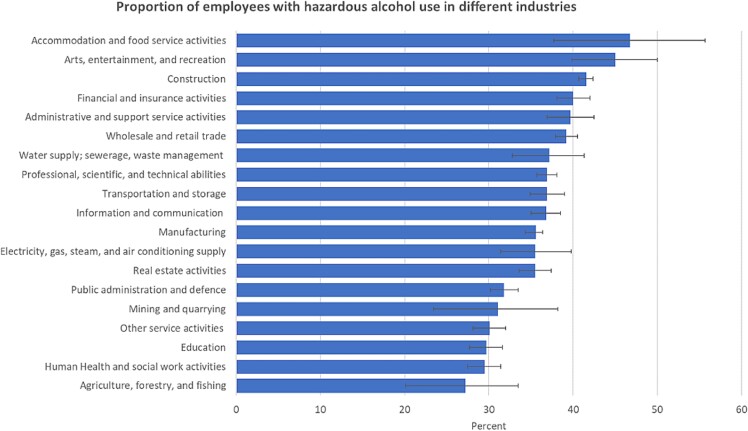
Proportion of employees with hazardous alcohol use in different industries

A total of 20 251 (37.2%) study participants were defined as having hazardous alcohol consumption. Individuals in accommodation/food service and arts/entertainment/recreation, construction, and financial/insurance activities had the highest prevalence; the lowest prevalence was found among individuals in industries such as education, human health/social work activities, and agriculture/forestry/fishing (Fig. 1).

The separate effects of each covariate, crude and adjusted, on the outcome of hazardous alcohol consumption are shown in Table 2. Increased odds were found among individuals who use nicotine and experience low work satisfaction and engagement, low social support, and heavy lifting in the workplace. Experience of workplace violence was associated with lower odds of hazardous alcohol consumption.

**Table 2:** Unadjusted and adjusted OR and 95% CI on the association between each potential risk factor and hazardous alcohol consumption

	Crude OR (95% CI)	Adjusted OR^a^ (95% CI)
Sex (ref: women)	1.06 (1.02, 1.10)	0.85 (0.82, 0.88)
Nicotine use	2.15 (2.07, 2.23)	2.11 (2.02, 2.19)
Poor general health	1.08 (0.98, 1.18)	1.02 (0.93, 1.12)
Feeling depressed	1.08 (0.97, 1.20)	0.94 (0.84, 1.07)
Feeling anxious	1.12 (1.02, 1.22)	1.04 (0.93, 1.15)
Low work satisfaction and engagement	1.15 (1.10, 1.19)	1.06 (1.01, 1.11)
Low social support	1.05 (1.01, 1.10)	1.02 (0.97, 1.07)
High workload	0.96 (0.91, 1.00)	0.98 (0.94, 1.03)
Workplace violence	0.77 (0.66, 0.89)	0.74 (0.63, 0.87)
Heavy lifting	1.34 (1.29, 1.39)	1.10 (1.06, 1.15)

^a^Adjusted OR: adjusted for year participating in HALU, age (continuous), sex, nicotine use, health (depression, anxiety, self-rated health), and psychosocial and physical work environment.

Compared to individuals working in education, most of the industries had an increased risk of hazardous alcohol consumption in the crude model (Table 3). More than 1.5-fold increased odds were found among individuals working in accommodation/food service (OR: 2.08, 95% CI 1.45–2.99), arts/entrainment/recreation (OR: 1.94, 95% CI 1.56–2.41), construction (OR: 1.69, 95% CI 1.53–1.86), financial/insurance activities (OR: 1.57, 95% CI 1.40–1.78), administrative/support service activities (OR: 1.55, 95% CI 1.34–1.80), and wholesale/retail trade (OR: 1.53, 95% CI 1.37–1.70). Adjusting for the sociodemographic factors had a marginal influence on the risk estimates. Differences in nicotine use (Model 2) explained a greater proportion of the increased risk in some of the industries but not all. Adjusting for mental and physical health as well as psychosocial work environments had limited effect on the estimates across all industries. Differences in physical work environment explained a greater proportion of the risk difference in a few industries, including construction. In the fully adjusted model, the increased odds of hazardous alcohol use remained for most industries, although slightly attenuated.

**Table 3:** Crude and adjusted ORs with 95% CI for the associations between industry and hazardous alcohol consumption. Industries are ordered by crude OR magnitude

Industry	Hazardous alcohol consumption total (%)	Crude	Model 1^a^	Model 2^b^	Model 3^c^	Model 4^d^	Model 5^e^	Model 6^f^
Accommodation and food service activities	59 (46.8)	2.08 (1.45, 2.99)	1.98 (1.38, 2.86)	1.62 (1.12, 2.34)	1.95 (1.36, 2.81)	1.98 (1.38, 2.85)	1.78 (1.23, 2.56)	1.54 (1.06, 2.23)
Arts, entertainment, and recreation	177 (45.0)	1.94 (1.56, 2.41)	1.98 (1.58, 2.46)	1.82 (1.46, 2.28)	1.97 (1.58, 2.46)	1.96 (1.57, 2.45)	1.90 (1.52, 2.37)	1.75 (1.40, 2.19)
Construction	5780 (41.6)	1.69 (1.53, 1.86)	1.57 (1.41, 1.73)	1.35 (1.21, 1.49)	1.57 (1.42, 1.74)	1.56 (1.41, 1.73)	1.44 (1.30, 1.60)	1.30 (1.17, 1.45)
Financial and insurance activities	1057 (40.0)	1.57 (1.40, 1.78)	1.52 (1.34, 1.72)	1.45 (1.28, 1.64)	1.54 (1.36, 1.73)	1.52 (1.34, 1.71)	1.55 (1.37, 1.75)	1.47 (1.30, 1.67)
Administrative and support service activities	482 (39.7)	1.55 (1.34, 1.80)	1.52 (1.31, 1.76)	1.34 (1.15, 1.56)	1.52 (1.31, 1.77)	1.51 (1.30, 1.75)	1.46 (1.26, 1.69)	1.33 (1.15, 1.55)
Wholesale and retail trade	2105 (39.2)	1.53 (1.37, 1.70)	1.50 (1.35, 1.67)	1.34 (1.20, 1.49)	1.51 (1.35, 1.68)	1.49 (1.34, 1.67)	1.44 (1.29, 1.61)	1.32 (1.18, 1.47)
Water supply; sewage, waste management, and remediation activities	195 (37.3)	1.41 (1.15, 1.72)	1.40 (1.15, 1.72)	1.20 (0.98, 1.47)	1.41 (1.16, 1.73)	1.41 (1.15, 1.72)	1.32 (1.08, 1.61)	1.18 (0.96, 1.45)
Professional, scientific, and technical abilities	2399 (36.9)	1.38 (1.24, 1.53)	1.31 (1.18, 1.45)	1.26 (1.13, 1.41)	1.32 (1.18, 1.47)	1.30 (1.17, 1.45)	1.31 (1.18, 1.46)	1.27 (1.14, 1.42)
Transportation and storage	769 (36.9)	1.38 (1.22, 1.57)	1.39 (1.22, 1.58)	1.24 (1.09, 1.41)	1.39 (1.22, 1.58)	1.37 (1.21, 1.56)	1.36 (1.19, 1.55)	1.23 (1.08, 1.41)
Information and communication	1065 (36.8)	1.38 (1.22, 1.55)	1.39 (1.23, 1.56)	1.34 (1.19, 1.51)	1.39 (1.24, 1.57)	1.38 (1.23, 1.56)	1.41 (1.25, 1.59)	1.34 (1.19, 1.52)
Manufacturing	2128 (35.6)	1.31 (1.18, 1.46)	1.33 (1.20, 1.49)	1.19 (1.07, 1.33)	1.34 (1.20, 1.49)	1.32 (1.18, 1.47)	1.26 (1.13, 1.41)	1.15 (1.03, 1.29)
Real estate activities	877 (35.5)	1.30 (1.15, 1.47)	1.31 (1.16, 1.49)	1.20 (1.06, 1.36)	1.32 (1.16, 1.49)	1.32 (1.17, 1.50)	1.27 (1.12, 1.44)	1.19 (1.05, 1.35)
Electricity, gas, steam, and air conditioning supply	198 (35.5)	1.30 (1.07, 1.58)	1.34 (1.10, 1.63)	1.19 (0.97, 1.45)	1.34 (1.10, 1.63)	1.34 (1.10, 1.63)	1.27 (1.04, 1.55)	1.16 (0.94, 1.41)
Public administration and defence, compulsory social security	968 (31.8)	1.10 (0.97, 1.24)	1.14 (1.01, 1.29)	1.06 (0.94, 1.20)	1.15 (1.02, 1.30)	1.15 (1.01, 1.29)	1.11 (0.98, 1.25)	1.07 (0.94, 1.20)
Mining and quarrying	50 (31.1)	1.07 (0.75, 1.51)	1.07 (0.76, 1.52)	0.87 (0.61, 1.24)	1.09 (0.77, 1.55)	1.06 (0.75 (1.50)	1.10 (0.77, 1.56)	0.88 (0.62, 1.26)
Other service activities	631 (30.1)	1.02 (0.89, 1.16)	1.06 (0.93, 1.21)	1.00 (0.87, 1.14)	1.07 (0.94, 1.22)	1.07 (0.94, 1.22)	1.03 (0.90, 1.18)	0.99 (0.87, 1.13)
Education (ref)	640 (29.7)	1.00	1.00	1.00	1.00	1.00	1.00	1.00
Human health and social work activities	622 (29.5)	0.99 (0.87, 1.13)	0.98 (0.86, 1.13)	0.90 (1.46, 2.28)	0.98 (0.85, 1.13)	0.99 (0.86, 1.14)	0.92 (0.80, 1.06)	0.90 (0.78, 1.04)
Agriculture, forestry, and fishing	49 (27.2)	0.88 (0.63, 1.24)	0.85 (0.60, 1.19)	0.76 (0.54, 1.08)	0.85 (0.60, 1.20)	0.85 (0.60, 1.20)	0.79 (0.56, 1.11)	0.74 (0.53, 1.06)

^a^Model 1: Adjusted for year participating in HALU, age (continuous), and sex.

^b^Model 2: Adjusted for model 1 and nicotine use.

^c^Model 3: Adjusted for model 1 and health (depression, anxiety, self-rated health).

^d^Model 4: Adjusted for model 1 and psychosocial work environment.

^e^Model 5: Adjusted for model 1 and physical work environment.

^f^Model 6: Adjusted for all explanatory factors simultaneously.

###  

#### Additional analysis

Excluding individuals who completed the HALU survey during the Covid-19 pandemic (*n* = 5515) demonstrated similar results as in the main analysis (Supplementary Table 5). Furthermore, a sensitivity analysis excluding individuals experiencing >4 weeks of sick leave (*n* = 2445) also demonstrated similar results as in the main analysis (Supplementary Table 6). After increasing the AUDIT-C cut-off, 11 031 (20.3%) were defined as having an AUD. Compared to individuals working in education, individuals working in construction demonstrated >2-fold increased risk of AUD in the crude model (Supplementary Table 7). After all adjustments, the risk differences remained slightly attenuated across almost all industries, which was like the results of the main analyses.

## Discussion

The current study aimed to examine differences in hazardous alcohol consumption across a wide range of industries in Sweden and to assess to what degree any such difference can be attributed to a differential distribution of nicotine use, health, and work-related factors among the individuals working in these industries. We found that in Sweden, accommodation/food, arts/entertainment/recreation arts/entertainment/ recreation, construction, and financial/ insurance activities were the industries with the highest prevalence of hazardous alcohol consumption. Compared to the education industry, an increased risk of hazardous alcohol consumption was found among most industries. Differences in nicotine use, health, and work-related factors explained a part but not all of the differences in risk.

In this study, the prevalence of hazardous drinkers was considerably higher among individuals working in industries of accommodation/food, arts/entertainment/recreation, construction, and financial/insurance activities, which is somewhat in line with previous research (Roche et al. 2015; Thompson and Pirmohamed 2021). In the current study, a larger proportion of females were found in the arts/entertainment/recreation, contradicting the previous assumption that much of the industry differences found are driven by males (Thompson and Pirmohamed 2021). On the opposite end of the spectrum, individuals in the education industry, human health/social activities, and agriculture/forestry/fishing reported the lowest prevalence of hazardous drinking, which is somewhat contradictory to previous research looking at alcohol-related mortality (Roche et al. 2015; Pulido et al. 2017). This shows the importance of considering variations in alcohol consumption across industries when researching alcohol-related injuries and mortality. The current study population comprised industries connected to occupational healthcare services, which could differ compared to those of the general working population regarding differences in who has and to what extent they can access occupational health services (Arbetsmiljöverket 2022; Feelgood 2023).

Supporting and extending previous research, we found an association between industry and self-reported measures of hazardous alcohol consumption in the current study (Roche et al. 2015; Tynan et al. 2017). Building upon previous research, we included a wide range of industries in the current study and found that industries not typically known to be high risk, such as arts/entertainment/recreation, financial/insurance activities, and wholesale/retail, had among the highest risk of hazardous alcohol consumption. Interestingly, individuals in the agriculture/forestry/fishing industry were less likely to report hazardous alcohol consumption compared to individuals working in education, which contradicts previous patterns found concerning alcohol-related mortality (Pulido et al. 2017). A recent Spanish study found >5-fold difference in alcohol-related mortality between the reference group of individuals in education and individuals in the agriculture/forestry/fishing industry (Pulido et al. 2017). There could be several reasons for this difference in patterns, such as differences in outcome measure, context, or study populations. The Spanish study examined a nationwide cohort of 16 million employees (Pulido et al. 2017), whereas our study studied those connected to occupational health services, something employees from the agriculture/forestry/fishing industry are known to be particularly unrepresented in (Arbetsmiljöverket 2022).

Extending previous research that investigated reasons for the industry differences in hazardous alcohol consumption (Pulido et al. 2017; James et al. 2021), we included several measures covering individuals’ nicotine use, health, and work-related factors. Nicotine use appeared to be of importance as these explained a larger part of the difference in risk of hazardous alcohol consumption in some of the industries, especially male-dominated industries, which were predominantly characterized by manual work, such as construction, manufacturing, and water supply/sewerage/waste management.

In line with previous research, we found that some work-related factors were risk factors for hazardous alcohol consumption, while other factors appeared to be protective (Zhu et al. 2011; Heikkilä et al. 2012; Roche et al. 2015; Tynan et al. 2017; Almroth et al. 2022). In the current study, hazardous alcohol use was less common among individuals who reported experiencing workplace violence (as seen in Table 2). In line with previous research, we found that workplace violence was more prevalent in female-dominated industries, where such violence is not typically considered part of the job (Nyberg et al. 2021). Furthermore, previous research suggests that there is a link between workplace violence and hazardous alcohol consumption for men but not for women, which could explain our findings (Rospenda et al. 2008). Differences in the investigated work-related factors only had a small influence on the risk estimates, which could be for several possible reasons. First, the study population comprised employees working in workplaces that used occupational health services, where health risks could be detected earlier compared to employees without connection to occupational health services (Ikonen et al. 2013). Additionally, the data on the work environment were not obtained from validated surveys. This lack of validation could have potentially impacted the results, as we cannot be certain that the questions in the HALU survey accurately measure what they intend to measure.

The results of the current study suggest a substantial variation in the prevalence of hazardous alcohol consumption across industries in Sweden. This finding highlights the importance of including detailed information about industries, as opposed to broad occupational categories to be able to identify and target interventions in high-risk working populations. The two main occupational groups in the three industries with the highest prevalence of hazardous alcohol consumption were service/care/shop sales workers and administration/communication workers. Despite being able to adjust for a broad range of potential individual and work-related covariates that could explain the excess risk of hazardous alcohol consumption, the elevated risk remained in most of the industries, suggesting that other factors are driving these differences that should be an avenue for future research. For example, findings from previous research suggest that alcohol availability and the alcohol habits of colleagues and managers can influence an employee’s alcohol consumption (Hodgins et al. 2009; Roche et al. 2015; Thørrisen et al. 2022). Several studies have found that workplace alcohol drinking norms are the strongest workplace factor influencing alcohol consumption and related harm, even after adjusting for various sociodemographic and other work-related factors (Hodgins et al. 2009; Thørrisen et al. 2022). Alcohol knowledge, attitudes, and workplace alcohol drinking norms vary across different industries, which could potentially explain the remaining risk differences (Roche et al. 2015; Roche et al. 2020),

### Strengths and limitations

Having a large sample size and data representing many different industries in Sweden, as opposed to focusing on only one industry, are major strengths of the current study. An additional strength was the wealth of information, which covered important dimensions of individual and work-related factors. The outcome hazardous alcohol consumption was defined using AUDIT-C, which is a well-renowned and validated scale to measure hazardous alcohol consumption.

A weakness, however, is the potential sampling bias of the study population given that it only represents a proportion of the Swedish workforce. Furthermore, several individuals were excluded due to missing information on key variables (i.e. industry and alcohol use) that might introduce some bias due to missing data. Using listwise deletion could potentially result in an overestimation or underestimation of the true association between industry and hazardous alcohol use. However, our sensitivity analyses using multiple imputations demonstrated similar results as in the main analysis. Furthermore, the included and excluded participants were generally quite similar on all covariates, except for heavy lifting when excluded individuals reported a higher prevalence. Nonetheless, although most industries are represented in the current study, the generalizability of the main results should be done with caution. Furthermore, the items related to health and the work environment included in the HALU survey are not validated. However, we performed a principal component analysis resulting in a three-factor solution with high Cronbach’s alpha, demonstrating high internal consistency for each factor. Due to the cross-sectional nature of the data, we cannot determine the temporal sequence and do not have information on any history of hazardous alcohol consumption.

## Conclusion

The results of this study suggest that the highest prevalence of hazardous alcohol consumption is found among individuals working in accommodation/food service, arts/entertainment/ recreation, and construction. While differences in nicotine use, health, and work environment account for some of the differences in the risk of hazardous alcohol consumption, the positive association persisted across most industries. This implies that other factors contribute to the excess risk, emphasizing the need for targeted prevention efforts within these industries.

## Supplementary Material

Supplementary_Figure_and_tabels_A_and_A_R2_agae077

## Data Availability

The data underlying this article were provided by Feelgood by permission. Data will be shared on request to the corresponding author with permission of Feelgood.
