# Supplementary material for: Hazardous alcohol consumption across different industries in Sweden: a pooled cross-sectional study
Source: Alcohol Alcohol. 2024 Nov 11;59(6):agae077. doi: 10.1093/alcalc/agae077 (PMC11554268; doi:10.1093/alcalc/agae077)
Supplement: Supplementary_Figure_and_tabels_A_and_A_R2_agae077 [file supplementary_figure_and_tabels_a_and_a_r2_agae077.docx]

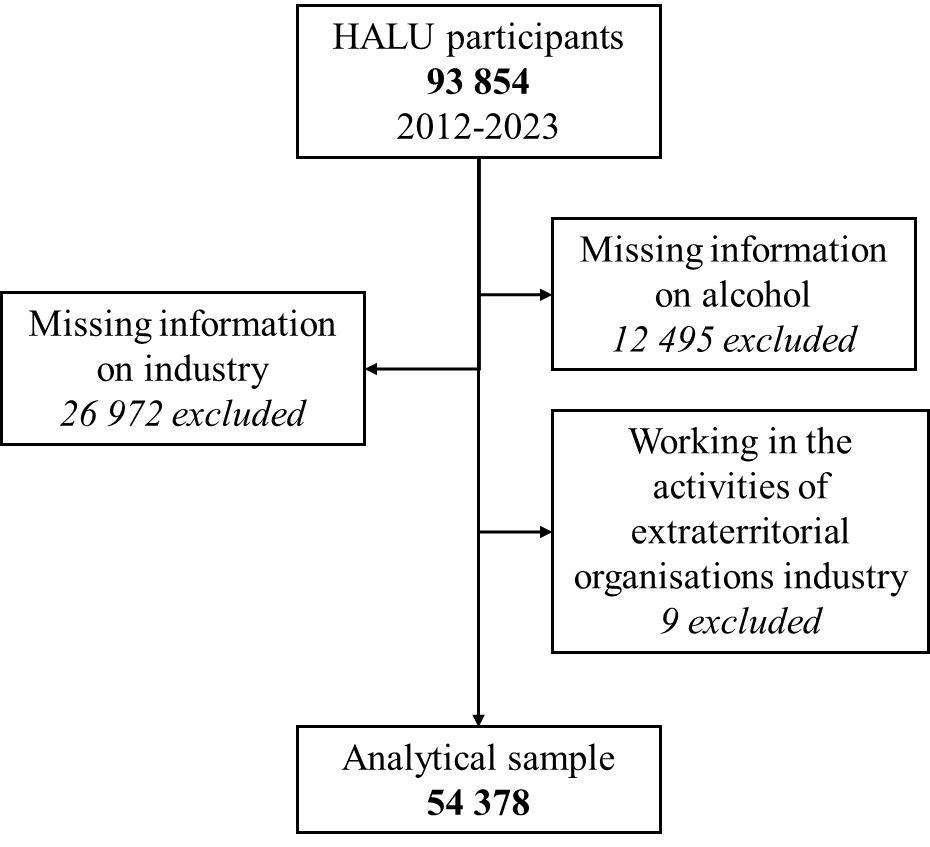


Supplementary Figure 1: Flow chart describing the selection process of the participants.

Supplementary Table 1. Characteristics of individuals included and excluded in the study population.

|  | **Included**  **n(%)** | **Excluded**  **n(%)** |
| --- | --- | --- |
| Total | 55 378 | 39 476 |
| Sex |  |  |
| Male | 35 271 (64.9) | 26 088 (66.1) |
| Female | 19 107 (35.1) | 13 379 (33.9) |
| Age (mean ±sd) | 41.7 ±12.0 | 42.7± 12.1 |
| Nicotine use | 15 700 (28.9) | 10 780 (27.3) |
| Poor general health | 2242 (4.1) | 1821 (4.6) |
| Feeling depressed | 1598 (2.9) | 1402 (3.6) |
| Feeling anxious | 2123 (3.9) | 1848 (4.7) |
| Low work satisfaction and engagement | 14 987 (27.6) | 10 043 (25.4) |
| Low social support | 13 390 (24.6) | 8922 (22.6) |
| High workload | 9468 (17.4) | 6089 (15.4) |
| Workplace violence | 797 (1.5) | 550 (1.4) |
| Heavy lifting | 15 431 (28.4) | 12 302 (31.2) |

Supplementary Table 2: Psychosocial work environment items included in each factor.

| **Factor** | **Items included** |
| --- | --- |
| 1. Work satisfaction and engagement | “My work feels meaningful.” |
|  | “I can decide how to plan and carry out my work.” |
|  | “I know which tasks are included in my position.” |
|  | “My tasks are appropriately varied.” |
|  | “I have a good balance between demand and resources.” |
|  | “My work engages me.” |
|  | “I can apply my knowledge in my work.” |
|  | “I receive the competence development I need in my work.” |
|  | “There is an openness to initiatives at my workplace.” |
| 1. Social support | “I enjoy work” |
|  | “The collaboration with colleagues is good.” |
|  | “I receive the support I need from my immediate supervisors.” |
|  | “I receive appreciation for my efforts at work.” |
|  | “I feel respected in my workplace.” |
|  | “If problems arise, I have someone at work to talk to.” |
| 1. Workload | “I feel stressed at work.” |
|  | “My job places too many demands on me.” |
|  | “I have so much to do that I feel inadequate.” |
|  | “I am bothered by urgency and tight time margins in my work.” |
|  | “I work under such time pressure that I don’t have time to talk to my colleagues, take coffee breaks or have lunch.” |
|  | “Changes within my company have affected me negatively.” |

Supplementary Table 3: MI individuals missing information on covariates, crude and adjusted ORs with 95% CI for the associations between industry and hazardous alcohol consumption. Industries were ordered by crude OR magnitude.

| **Industry** | **Hazardous alcohol consumption %** | **Crude** | **Model 1** | **Model 2** | **Model 3** | **Model 4** | **Model 5** | **Model 6** |
| --- | --- | --- | --- | --- | --- | --- | --- | --- |
| Accommodation and food service activities | 46.7% | 2.08  (1.44, 2.99) | 1.98  (1.37, 2.85) | 1.62  (1.12, 2.36) | 1.96  (1.36, 2.82) | 1.97  (1.27, 2.83) | 1.87  (1.20, 2.69) | 1.59  (1.09, 2.31) |
| Arts, entertainment, and recreation | 44.7% | 1.93  (1.53, 2.43) | 1.96  (1.55, 2.47) | 1.79  (1.42, 2.26) | 1.96  (1.55, 2.47) | 1.95  (1.54, 2.46) | 1.92  (1.52, 2.42) | 1.78  (1.41, 2.24) |
| Construction | 41.3% | 1.66  (1.50, 1.84) | 1.58  (1.42, 1.75) | 1.35  (1.21, 1.51) | 1.58  (1.42, 1.75) | 1.57  (1.42, 1.75) | 1.51  (1.26, 1.68) | 1.33  (1.19, 1.49) |
| Financial and insurance activities | 39.7% | 1.56  (1.36, 1.79) | 1.51  (1.32, 1.73) | 1.44  (1.25, 1.64) | 1.52  (1.32, 1.74) | 1.51  (1.32, 1.73) | 1.53  (1.33, 1.75) | 1.45  (1.26, 1.66) |
| Administrative and support service activities | 39.1% | 1.55  (1.35, 1.78) | 1.53  (1.33, 1.76) | 1.35  (1.17, 1.56) | 1.53  (1.33, 1.76) | 1.52  (1.32, 1.75) | 1.50  (1.30, 1.72) | 1.34  (1.16, 1.55) |
| Wholesale and retail trade | 39.1% | 1.52  (1.35, 1.70) | 1.50  (1.34, 1.68) | 1.34  (1.19, 1.50) | 1.50  (1.34, 1.69) | 1.50  (1.33, 1.68) | 1.47  (1.31, 1.65) | 1.32  (1.18, 1.49) |
| Water supply; sewerage, waste management and remediation activities | 36.8% | 1.40  (1.10, 1.77) | 1.41  (1.11, 1.78) | 1.20  (0.94, 1.54) | 1.41  (1.11, 1.79) | 1.40  (1.10, 1.78) | 1.36  (1.07, 1.73) | 1.19  (0.93, 1.52) |
| Professional, scientific, and technical abilities | 36.7% | 1.37  (1.23, 1.51) | 1.30  (1.17, 1.45) | 1.26  (1.13, 1.40) | 1.31  (1.18, 1.45) | 1.30  (1.17, 1.45) | 1.31  (1.18, 1.45) | 1.26  (1.13, 1.40) |
| Transportation and storage | 36.6% | 1.37  (1.21, 1.56) | 1.38  (1.21, 1.57) | 1.23  (1.08, 1.40) | 1.38  (1.21, 1.57) | 1.37  (1.20, 1.56) | 1.36  (1.20, 1.55) | 1.23  (1.08, 1.40) |
| Information and communication | 36.5% | 1.37  (1.21, 1.54) | 1.38  (1.22, 1.56) | 1.33  (1.18, 1.51) | 1.38  (1.22, 1.56) | 1.37  (1.22, 1.55) | 1.39  (1.30, 2.69) | 1.34  (1.18, 1.51) |
| Manufacturing | 35.3% | 1.31  (1.17, 1.45) | 1.34  (1.21, 1.49) | 1.20  (1.07, 1.34) | 1.34  (1.21, 1.49) | 1.33  (1.20, 1.48) | 1.30  (1.17, 1.45) | 1.18  (1.06, 1.32) |
| Real estate activities | 35.3% | 1.29  (1.14, 1.46) | 1.31  (1.16, 1.48) | 1.19  (1.05, 1.35) | 1.31  (1.16, 1.49) | 1.31  (1.16, 1.49) | 1.29  (1.14, 1.46) | 1.19  (1.05, 1.35) |
| Electricity, gas, steam, and air conditioning supply | 34.8% | 1.28  (1.04, 1.57) | 1.35  (1.10, 1.66) | 1.20  (0.97, 1.47) | 1.34  (1.10, 1.65) | 1.34  (1.09, 1.64) | 1.31  (1.06, 1.61) | 1.18  (0.96, 1.45) |
| Public administration and defence, compulsory social security | 31.7% | 1.09  (0.97, 1.24) | 1.13  (0.99, 1.27) | 1.05  (0.92, 1.19) | 1.13  (1.00, 1.28) | 1.12  (0.99, 1.27) | 1.10  (0.97, 1.25) | 1.04  (0.92, 1.18) |
| Mining and quarrying | 29.9% | 1.04  (0.72, 1.50) | 1.07  (0.74, 1.55) | 0.88  (0.60, 1.29) | 1.07  (0.74, 1.56) | 1.06  (0.73, 1.54) | 1.08  (0.74, 1.57) | 0.88  (0.61, 1.29) |
| Other service activities | 29.9% | 1.02  (0.90, 1.16) | 1.07  (0.94, 1.22) | 1.00  (0.87, 1.14) | 1.07  (0.94, 1.22) | 1.07  (0.94, 1.22) | 1.05  (0.92, 1.20) | 1.00  (0.88, 1.14) |
| Education (ref) | 29.7% | 1.00 | 1.00 | 1.00 | 1.00 | 1.00 | 1.00 | 1.00 |
| Human Health and social work activities | 29.4% | 0.99  (0.87, 1.13) | 1.02  (0.89, 1.16) | 0.94  (0.82, 1.08) | 1.02  (0.89, 1.16) | 1.02  (0.89, 1.17) | 0.99  (0.86, 1.13) | 0.93  (0.82, 1.07) |
| Agriculture, forestry, and fishing | 27.6% | 0.86  (0.61, 1.23) | 0.85  (0.60, 1.22) | 0.76  (0.53, 1.10) | 0.85  (0.60, 1.22) | 0.85  (0.60, 1.21) | 0.83  (0.58, 1.18) | 0.75  (0.52, 1.09) |

Model 1: Adjusted for year participating in HALU, age (continuous) and sex.

Model 2: Adjusted for model 1 and nicotine use.

Model 3: Adjusted for model 1 and health (depression, anxiety, self-rated health).

Model 4: Adjusted for model 1 and psychosocial work environment.

Model 5: Adjusted for model 1 and physical work environment.

Model 6: Adjusted for all explanatory factors simultaneously.

Supplementary Table 4: Complete case analysis excluding 1194 individuals missing information on covariates, crude and adjusted ORs with 95% CI for the associations between industry and hazardous alcohol consumption. Industries were ordered by crude OR magnitude.

| **Industry** | **Hazardous alcohol consumption total (%)** | **Crude** | **Fully adjusted** |
| --- | --- | --- | --- |
| Accommodation and food service activities | 58 (47.2) | 2.11 (1.46, 3.04) | 1.55 (1.06, 2.25) |
| Arts, entertainment, and recreation | 171 (44.5) | 1.90 (1.52, 2.37) | 1.74 (1.39, 2.18) |
| Construction | 5617 (41.6) | 1.69 (1.53, 1.86) | 1.29 (1.16, 1.44) |
| Administrative and support service activities | 477 (40.2) | 1.59 (1.37, 1.85) | 1.35 (1.16, 1.57) |
| Financial and insurance activities | 1040 (39.8) | 1.57 (1.39, 1.77) | 1.46 (1.29, 1.65) |
| Wholesale and retail trade | 2074 (39.4) | 1.53 (1.38, 1.71) | 1.31 (1.17, 1.47) |
| Water supply; sewerage, waste management and remediation activities | 193 (37.6) | 1.42 (1.16, 1.74) | 1.18 (0.96, 1.45) |
| Transportation and storage | 758 (37.0) | 1.39 (1.22, 1.58) | 1.23 (1.07, 1.40) |
| Professional, scientific, and technical abilities | 2342 (36.8) | 1.37 (1.24, 1.53) | 1.26 (1.13, 1.40) |
| Information and communication | 1037 (36.4) | 1.35 (1.20, 1.53) | 1.32 (1.17, 1.49) |
| Manufacturing | 2069 (35.5) | 1.30 (1.17, 1.45) | 1.14 (1.02, 1.27) |
| Real estate activities | 864 (35.3) | 1.29 (1.14, 1.46) | 1.18 (1.04, 1.34) |
| Electricity, gas, steam, and air conditioning supply | 191 (35.1) | 1.28( 1.05, 1.56) | 1.14 (0.93, 1.40) |
| Public administration and defence, compulsory social security | 936 (31.9) | 1.11 (0.98, 1.25) | 1.07 (0.94, 1.21) |
| Mining and quarrying | 50 (31.1) | 1.06 (0.75, 1.51) | 0.87 (0.61, 1.24) |
| Other service activities | 620 (30.2) | 1.02 (0.99 (1.17) | 0.99 (0.87, 1.13) |
| Education (ref) | 629 (29.7) | 1.00 | 1.00 |
| Human Health and social work activities | 614 (29.6) | 0.99 (0.87, 1.13) | 0.89 (0.77, 1.03) |
| Agriculture, forestry, and fishing | 49 (28.3) | 0.93 (0.66, 1.32) | 0.77 (0.54, 1.10) |

Fully adjusted: Adjusted for year participating in HALU, age (continuous), sex, nicotine use, health (depression, anxiety, self-rated health), psychosocial and physical work environment.

Supplementary Table 5: Additional analyses excluding individuals participating in 2020 or 2021 (n= 5515), crude and adjusted ORs with 95% CI for the associations between industry and hazardous alcohol consumption. Industries are ordered by crude OR magnitude.

| **Industry** | **Hazardous alcohol consumption total (%)** | **Crude** | **Fully adjusted** |
| --- | --- | --- | --- |
| Accommodation and food service activities | 56 (45.9) | 2.01 (1.39, 2.91) | 1.49 (1.02, 2.17) |
| Arts, entertainment, and recreation | 165 (45.1) | 1.95 (1.55, 2.44) | 1.77 (1.41, 2.23) |
| Construction | 4861 (42.1) | 1.72 (1.56, 1.90) | 1.33 (1.20, 1.48) |
| Financial and insurance activities | 981 (40.2) | 1.59 (1.41, 1.80) | 1.48 (1.30, 1.68) |
| Administrative and support service activities | 456 (39.6) | 1.55 (1.34, 1.80) | 1.32 (1.13, 1.54) |
| Wholesale and retail trade | 1951 (39.2) | 1.52 (1.37, 1.70) | 1.31 (1.17, 1.47) |
| Water supply; sewerage, waste management and remediation activities | 187 (37.9) | 1.45 (1.18, 1.78) | 1.22 (0.99, 1.51) |
| Information and communication | 989 (36.9) | 1.38 (1.23, 1.56) | 1.35 (1.19, 1.53) |
| Professional, scientific, and technical abilities | 2163 (36.6) | 1.37 (1.23, 1.53) | 1.26 (1.13, 1.54) |
| Transportation and storage | 712 (36.5) | 1.36 (1.20, 1.56) | 1.21 (1.06, 1.38) |
| Real estate activities | 788 (36.1) | 1.34 (1.18, 1.52) | 1.22 (1.07, 1.39) |
| Manufacturing | 1915 (35.5) | 1.31 (1.17, 1.46) | 1.15 (1.03, 1.29) |
| Electricity, gas, steam, and air conditioning supply | 183 (34.7) | 1.26 (1.03, 1.54) | 1.14 (0.93, 1.40) |
| Public administration and defence, compulsory social security | 919 (31.8) | 1.11 (0.98, 1.25) | 1.07 (0.95, 1.21) |
| Mining and quarrying | 41 (30.6) | 1.05 (0.72, 1.53) | 0.88 (0.60, 1.30) |
| Education (ref) | 635 (29.7) | 1.00 | 1.00 |
| Other service activities | 557 (29.5) | 0.99 (0.87, 1.14) | 0.96, 0.84, 1.11) |
| Human Health and social work activities | 556 (29.5) | 0.99 (0.86, 1.14) | 0.90 (0.77, 1.04) |
| Agriculture, forestry, and fishing | 43 (26.9) | 0.87 (0.61, 1.25) | 0.73 (0.50, 1.06) |

Fully adjusted: Adjusted for year participating in HALU, age (continuous), sex, nicotine use, health (depression, anxiety, self-rated health), psychosocial and physical work environment.

Supplementary Table 6: Additional analyses excluding individuals with more than four weeks of sick leave (n= 3585), crude and adjusted ORs with 95% CI for the associations between industry and hazardous alcohol consumption. Industries are ordered by crude OR magnitude.

| **Industry** | **Hazardous alcohol consumption total (%)** | **Crude** | **Fully adjusted** |
| --- | --- | --- | --- |
| Accommodation and food service activities | 59 (46.8) | 2.08 (1.45, 2.99) | 1.54 (1.06, 2.23) |
| Arts, entertainment, and recreation | 172 (45.6) | 1.98 (1.59, 2.48) | 1.83 (1.46, 2.29) |
| Construction | 5518 (41.6) | 1.69 (1.53, 1.87) | 1.31 (1.18, 1.56) |
| Financial and insurance activities | 1023 (40.1) | 1.58 (1.40, 1.79) | 1.48 (1.30, 1.68) |
| Wholesale and retail trade | 2030 (39.5) | 1.54 (1.38, 1.72) | 1.33 (1.19, 1.49) |
| Administrative and support service activities | 461 (39.5) | 1.54 (1.33, 1.80) | 1.31 (1.13, 1.53) |
| Transportation and storage | 735 (37.3) | 1.41 (1.23, 1.60) | 1.25 (1.09, 1.43) |
| Professional, scientific, and technical abilities | 2329 (37.1) | 1.40 (1.26, 1.56) | 1.29 (1.15, 1.44) |
| Information and communication | 1036 (37.0) | 1.39 (1.23, 1.57) | 1.36 (1.20, 1.54) |
| Water supply; sewerage, waste management and remediation activities | 179 (36.2) | 1.34 (1.09, 1.65) | 1.14 (0.92, 1.41) |
| Manufacturing | 2029 (35.7) | 1.31 (1.18, 1.46) | 1.16 (1.04, 1.30) |
| Real estate activities | 832 (35.5) | 1.30 (1.14, 1.47) | 1.19 (1.04, 1.35) |
| Electricity, gas, steam, and air conditioning supply | 190 (35.4) | 1.30 (1.06, 1.58) | 1.17 (0.96, 1.44) |
| Public administration and defence, compulsory social security | 923 (32.0) | 1.11 (0.98, 1.26) | 1.07 (0.95, 1.21) |
| Mining and quarrying | 50 (31.1) | 1.07 (0.75, 1.51) | 0.88 (0.62, 1.26) |
| Other service activities | 606 (30.7) | 1.05 (0.92, 1.20) | 1.02 (0.89, 1.17) |
| Human Health and social work activities | 586 (30.1) | 1.02 (0.89, 1.16) | 0.92 (0.79, 1.06) |
| Education (ref) | 613 (29.7) | 1.00 | 1.00 |
| Agriculture, forestry, and fishing | 47 (27.3) | 0.89 (0.63, 1.26) | 0.76 (0.53, 1.08) |

Fully adjusted: Adjusted for year participating in HALU, age (continuous), sex, nicotine use, health (depression, anxiety, self-rated health), psychosocial and physical work environment.

Supplementary Table 7: Crude and adjusted ORs with 95% CI for the associations between industry and alcohol use disorders. Industries are ordered by crude OR magnitude.

| **Industry** | **Alcohol use disorders**  **total (%)** | **Crude** | **Fully adjusted** |
| --- | --- | --- | --- |
| Construction | 3569 (25.7) | 2.11 (1.86, 2.40) | 1.30 (1.14, 1.49) |
| Arts, entertainment, and recreation | 95 (24.1) | 1.95 (1.50, 2.53) | 1.77 (1.35, 2.30) |
| Accommodation and food service activities | 29 (23.0) | 1.83 (1.19, 2.81) | 1.20 (0.77, 1.87) |
| Administrative and support service activities | 275 (22.6) | 1.79 (1.49, 2.14) | 1.37 (1.14, 1.65) |
| Wholesale and retail trade | 1180 (22.0) | 1.72 (1.50, 1.98) | 1.30 (1.13, 1.50) |
| Financial and insurance activities | 535 (20.2) | 1.55 (1.33, 1.81) | 1.42 (1.21, 1.66) |
| Manufacturing | 1157 (19.4) | 1.47 (1.28, 1.68) | 1.10 (0.95, 1.27) |
| Electricity, gas, steam, and air conditioning supply | 108 (19.4) | 1.47 (1.15, 1.87) | 1.13 (0.88, 1.45) |
| Information and communication | 557 (19.3) | 1.46 (1.23, 1.70) | 1.34 (1.14, 1.56) |
| Water supply; sewerage, waste management and remediation activities | 101 (19.3) | 1.46 (1.14, 1.88) | 1.05 (0.81, 1.36) |
| Transportation and storage | 393 (18.8) | 1.42 (1.20, 1.67) | 1.17 (0.99, 1.38) |
| Professional, scientific, and technical abilities | 1221 (18.8) | 1.41 (1.23, 1.62) | 1.23 (1.07, 1.41) |
| Real estate activities | 432 (17.5) | 1.29 (1.10, 1.52) | 1.12 (0.95, 1.32) |
| Mining and quarrying | 25 (15.5) | 1.12 (0.72, 1.75) | 0.83 (0.53, 1.31) |
| Other service activities | 313 (14.9) | 1.07 (0.90, 1.27) | 1.07 (0.90, 1.28) |
| Agriculture, forestry, and fishing | 26 (14.4) | 1.03 (0.67, 1.59) | 0.73 (0.47, 1.14) |
| Public administration and defence, compulsory social security | 428 (14.0) | 1.00 (0.85, 1.17) | 1.00 (0.85, 1.18) |
| Education (ref) | 303 (14.1) | 1.00 | 1.00 |
| Human Health and social work activities | 284 (13.5) | 0.95 (0.80, 1.13) | 0.94 (0.78, 1.13) |

Fully adjusted: Adjusted for year participating in HALU, age (continuous, sex, nicotine use, health (depression, anxiety, self-rated health), psychosocial and physical work environment.
